# Fatal *Vibrio vulnificus* Infection Associated with Eating Raw Oysters, New Caledonia

**DOI:** 10.3201/eid1701.100603

**Published:** 2011-01

**Authors:** Cecile Cazorla, Aurelie Guigon, Martine Noel, Marie-Laure Quilici, Flore Lacassin

**Affiliations:** Author affiliations: New Caledonian Territorial Hospital Internal Medecine Unit, Noumea, New Caledonia (C. Cazorla, F. Lacassin);; Institut Pasteur, Noumea (A. Guigon); Direction des Affaires Sanitaires et Sociales de Nouvelle Caledonie, Noumea (M. Noel);; Institut Pasteur, Paris, France (M.-L. Quilici)

**Keywords:** Bacteria, foodborne infections, shellfish, South Pacific, sepsis, Vibrio vulnificus, necrotizing cellulitis, liver disease, New Caledonia, letter

**To the Editor**: The bacterium *Vibrio vulnificus* is a marine flora saprophyte that can cause necrotic skin infection and septicemia in humans who eat shellfish. Symptoms of septicemia (mortality rate >50%) have been described mostly in Florida and Japan among persons who ate raw filter-feeding shellfish when seawater temperatures are >20°C (*1*).

*V. vulnificus*–related septicemia introduced through the digestive system appears within 7 days after ingestion (*2*). Clinical signs and symptoms include fever, collapse, and metastatic necrotic skin lesions. We report 3 patients from New Caledonia who died after *V. vulnificus* infection, which they probably acquired by eating contaminated oysters. These patients were hospitalized during February–May 2008 at Noumea Hospital (Noumea, New Caledonia).

Patient 1 was a 51-year-old man with fever, muscle pains, bleeding gums, and a history of alcohol abuse; within 48 hours after symptom onset, he died of septic shock, with diffuse ecchymoses and purpura. Patient 2 was a 67-year-old woman with no known concurrent conditions who was admitted to the hospital with chills, diarrhea, and vomiting; septic shock developed, with painful erythematous plaques on the lower limbs becoming foamy, confluent, and necrotic. Patient 3 was a 74-year-old woman with untreated lupus who was hospitalized with lower-limb edema, hypotension, hypothermia, and erythematous skin lesions. All 3 patients received cephalosporins but died of multiple organ failure within 12 hours after hospital admission.

Peripheral blood aerobic–anaerobic samples were taken from all patients, stored in BacT/Alert FA vials (bioMérieux, Marcy-l’Etoile, France), and incubated in the BacT/Alert 3D system (bioMérieux). Curved mobile gram-negative bacilli were isolated from blood samples cultured on conventional media without additional salt within 24 h after incubation at 37°C in a 5% CO_2_-enriched atmosphere. *V. vulnificus* was identified through the Vitek2 system (bioMérieux) and confirmed by using the Api 20E system (bioMérieux).

Strains were sent to the Centre National de Reference des Vibrions et du Choléra, (Institut Pasteur, Paris, France), which by PCR confirmed the gene encoding virulence-associated hemolysin, a species-specific marker (*3*). Molecular typing by pulsed-field gel electrophoresis was performed to assess possible clonality of the strains.

Several studies have shown the genomic diversity among environmental and clinical *V. vulnificus* isolates. The use of genotyping methods has identified >100 *V. vulnificus* strains in a single oyster (*4*) and notable heterogeneity among clinical isolates from multiple patients, even if a unique pathogenic strain causes the infection in each patient. Thus, *V. vulnificus* infections within a large population at risk may result from rare events controlled more by the host than by the bacterial strain (*5*).

Pulsed-field gel electrophoresis genotype analysis enabled us to divide the strains into 2 groups. One group included the isolate from patient 1, and the other group included isolates from patients 2 and 3, which despite having slightly different *Not*I and *Sfi*I patterns reflecting genetic rearrangement, clearly belonged to a single clone. Isolation of strains with such a high degree of homogeneity is not common, raising the question of the existence of *V. vulnificus* clones that are particularly virulent or adapted to humans. Currently, however, reliable markers for determining *V. vulnificus* virulence do not exist. Thus, no genotyping system is likely to be useful for rapidly identifying strains that affect public health (*6*). *V. vulnificus*–related analysis requires the assumption that all strains are virulent.

Epidemiologic information collected from patients’ families indicated recent consumption of raw oysters. Two of the 3 cases occurred within a short time frame and were associated with eating local oysters harvested on the west coast of New Caledonia.

The literature mentions few cases of *V. vulnificus* infection in the South Pacific. Cases described were isolated, rarely fatal, and involved infection through the skin (*7**–**10*). The *V. vulnificus* infections we report may be related to the emergence of a new clone or to changes in the climate or environmental conditions. New Caledonia experienced unusual weather conditions during the first half of 2008 (heavy rains and exceptionally high temperatures). These specific conditions may have favored higher sea surface temperatures, lower salinity, increased turbidity, and subsequent multiplication of *V. vulnificus* in seawater.

A range of projects were implemented to train practitioners to recognize potential *V. vulnificus* infections. Local health authorities issued criteria for defining suspected cases of *V. vulnificus* infection and recommendations for early medical care of patients with clinical symptoms. Methods of detecting the bacterium in human and animal health laboratories were improved, particularly by the systematic use of selective media in the event of suspected clinical *V. vulnificus* infection and standardized reporting of *V. vulnificus* isolation. Preventive measures, such as improving microbial surveillance and warning consumers about risks associated with eating raw seafood, are essential to help reduce the risk for *V. vulnificus*–induced illness.
